# Supplementary material for: Pelargonium graveolens: Towards In-Depth Metabolite Profiling, Antioxidant and Enzyme-Inhibitory Potential
Source: Plants (Basel). 2024 Sep 19;13(18):2612. doi: 10.3390/plants13182612 (PMC11434692; doi:10.3390/plants13182612)
Supplement: Supplementary file 1 [file plants-13-02612-s001.zip › plants-3173632-supplementary.pdf]

Pelargonium graveolens: Towards In-Depth Metabolite Profiling, Antioxidant and Enzyme-Inhibitory Potential

Reneta Gevrenova [1,*], Gokhan Zengin [2], Vessela Balabanova [1], Anna Szakiel [3] and Dimitrina Zheleva-Dimitrova [1]

[1] Department of Pharmacognosy, Faculty of Pharmacy, Medical University-Sofia, 2 Dunav Str., 1000 Sofia, Bulgaria; vbalabanova@pharmfac.mu-sofia.bg (V.B.); dzheleva@pharmfac.mu-sofia.bg (D.Z.-D.)
[2] Department of Biology, Science Faculty, Selcuk University, Konya 42130, Turkey; gokhanzengin@selcuk.edu.tr
[3] Department of Plant Biochemistry, Faculty of Biology, University of Warsaw, 1 Miecznikowa Street, 02-096 11 Warsaw, Poland; a.szakiel@uw.edu.pl
* Correspondence: rgervrenova@pharmfac.mu-sofia.bg

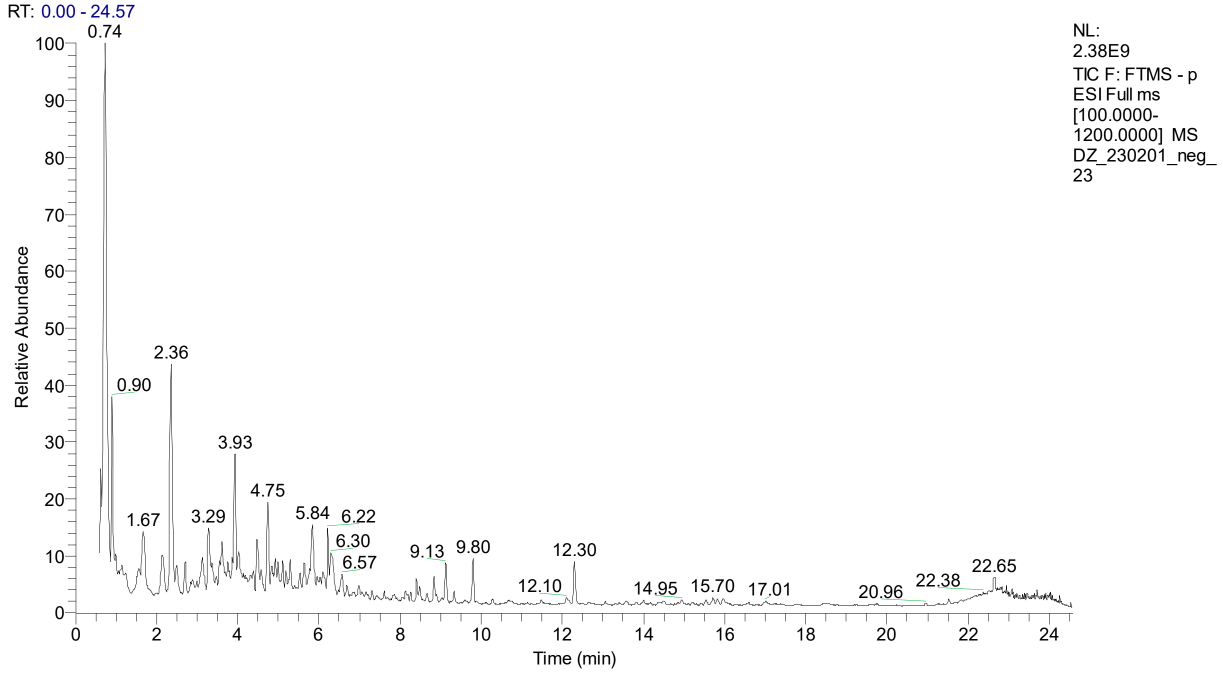

Figure S1. Total ion chromatogram (TIC) of methanol-aqueous extract of *Pelargonium graveolens*.

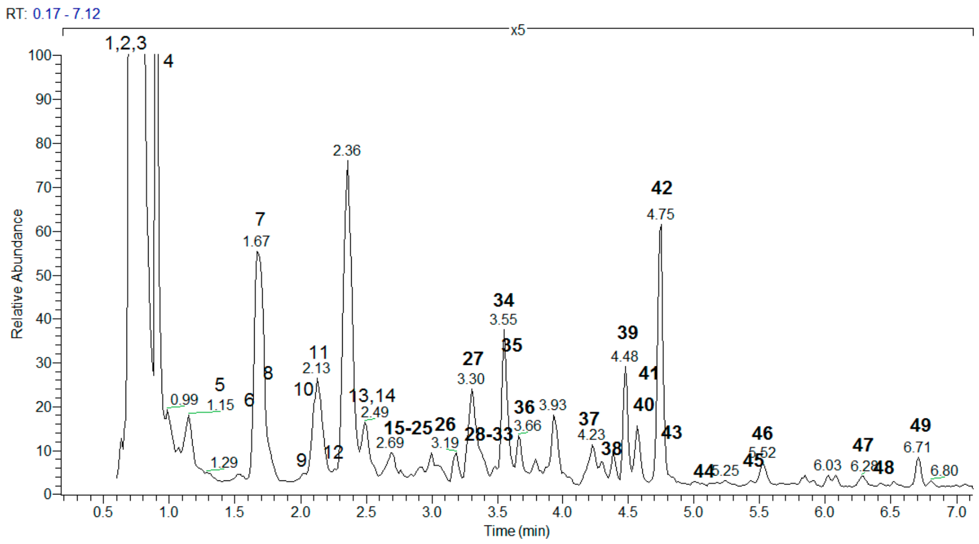

Figure S2. Extracted ion chromatogram of hydroxybenzoic, hydroxycinnamic acids and their glycosides, coumarins and aliphatic acids (amplified region x5) (for numbers and fragmentation patterns, see Table 1)

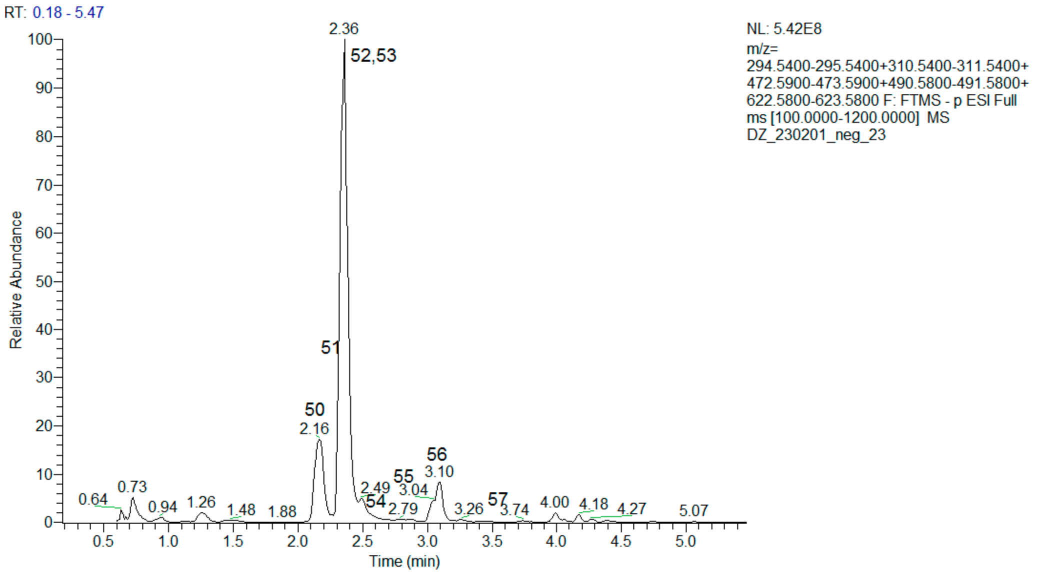

Figure S3. Extracted ion chromtogram of acyltartaric acids (for numbers and fragmentation patterns, see Table 1).

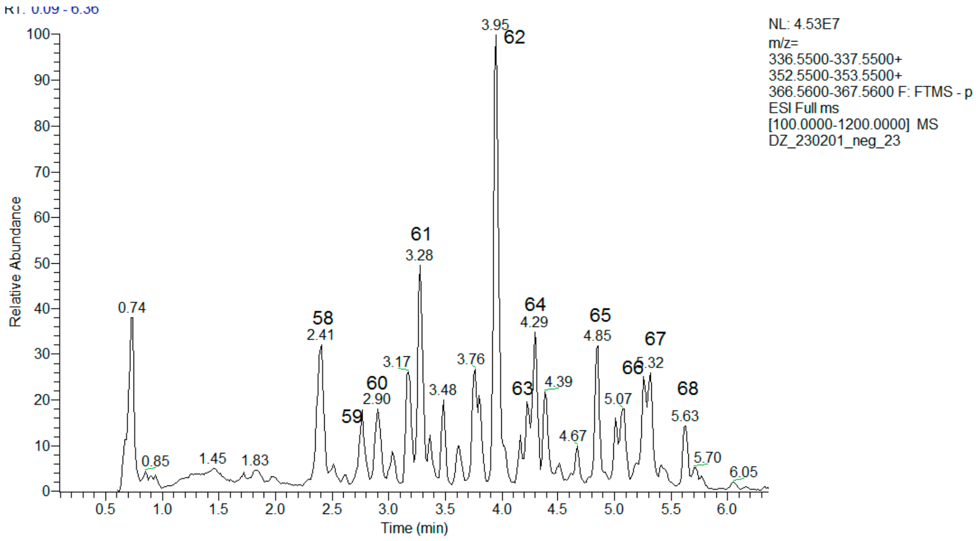

Figure S4. Extracted ion chromtogram of acylcitric/acylisocitric acids (for numbers and fragmentation patterns, see Table 1).

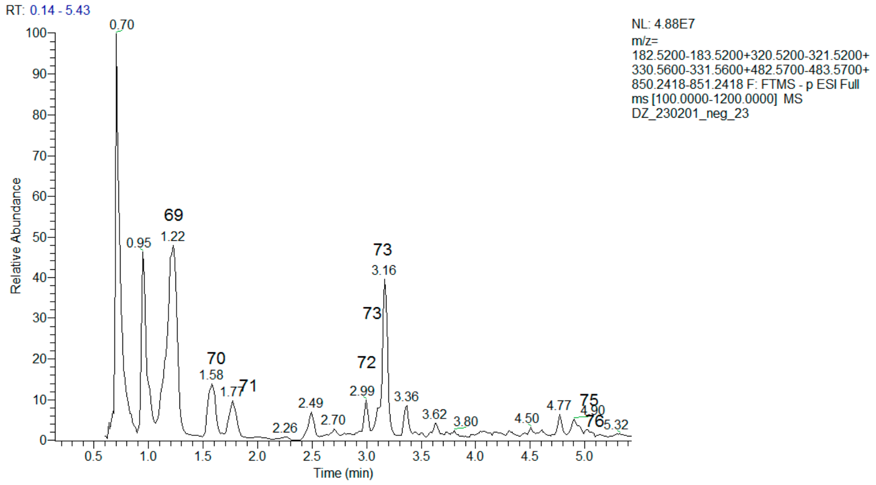

Figure S5. Extracted ion chromtogram of gallic acid derivatives (for numbers and fragmentation patterns, see Table 1).

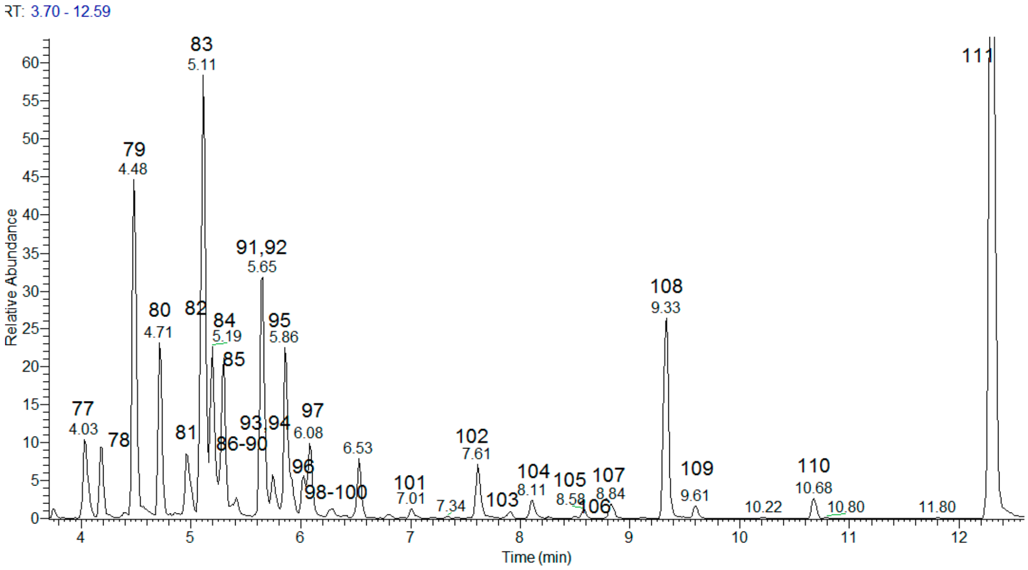

Figure S6. Extracted ion chromatogram of flavonoids (amplified region between 3.70 and 13 min) (for numbers and fragmentation patterns, see Table 1).

Table S1. Secondary metabolites in *Pelargonium graveolens* methanol-aqueous extracts

№	Identified/tentatively annotated compound	Molecular formula	Exact mass [M-H]$^-$	Fragmentation pattern in (-) ESI-MS/MS	t_R (min)	Δ ppm	Identification confidence level (Çiçek et al., 2024)
**Hydroxybenzoic, hydroxycinnamic acids and their glycosides, coumarins and aliphatic acids**							
1.	tartaric acid	$C_4H_6O_6$	149.0092	149.0079 (32.3), 130.0969 (1.6), 105.0179 (7.0), 103.0022 (9.1), 87.0071 (96.6), 72.9915 (100)	0.70	-8.531	D1
2.	mallic acid	$C_4H_6O_5$	133.0143	133.0130 (15.7), 115.0021 (97.2), 71.0122 (100)	0.74	-9.221	D1
3.	citric/isocitric acid	$C_6H_8O_7$	191.0197	191.0089 (9.2), 173.0079 (7.2), 154.9973 (5.2), 147.0289 (0.6), 129.0170 (8.0), 111.0072 (100), 85.0279 (35.9)	0.75	-4.271	D1
4.	citric/isocitric acid	$C_6H_8O_7$	191.0197	191.0087 (8.5), 173.0088 (1.5), 154.9970 (1.0), 147.0287 (0.4), 129.0178 (7.0), 111.0072 (100), 87.0071 (42.1), 85.0279 (27.7)	0.90	-5.318	D1
5.	gallic acid	$C_7H_6O_5$	169.0143	169.0130 (33.0), 125.0229 (100), 107.0123 (0.9)	1.15	-7.257	B
6.	protocatechuic acid-*O*-dihexoside	$C_{19}H_{26}O_{14}$	477.1250	477.1249 (100), 153.0183 (12.2). 152.0103 (28.4), 123.0073 (1.7). 109.0284 (5.5), 108.0202 (26.2)	1.52	-0.207	D1
7.	protocatechuic acid-*O*-hexoside 1	$C_{13}H_{16}O_9$	315.0727	315.0723 (100), 153.0181 (27.2), 152.0101 (59.9), 123.0074 (2.9), 109.0286 (11.2), 108.0201 (93.1)	1.67	0.459	D1
8.	vanillic acid-*O*-hexoside 1	$C_{14}H_{18}O_9$	329.0875	329.0894 (2.0),167.0337 (100), 152.0102 (21.0), 123.0437 (13.3), 108.0201 (37.3)	1.77	-0.381	D1
9.	protocatechuic acid	$C_7H_6O_4$	153.0181	153.0180 (14.9), 109.0279 (100), 91.0172 (1.4)	2.03	-8.639	B
10.	protocatechuic acid-*O*-hexoside 2	$C_{13}H_{16}O_9$	315.0727	315.0724 (100), 153.0180 (61.7), 109.0280 (74.0)	2.10	0.650	D1
11.	hydroxybenzoic acid-*O*-hexoside 1	$C_{13}H_{16}O_8$	299.0778	299.0784 (0.3), 137.0229 (100), 109.0280 (0.3), 93.0329 (38.1)	2.15		D1

12.	syringic acid-*O*-hexoside	$C_{15}H_{20}O_{10}$	359.0985	359.0990 (7.7), 197.0440 (100), 182.0210 (20.3), 166.9974 (8.2), 153.0544 (15.5), 138.0309 (26.4), 123.0073 (28.9)	2.26	1.671	D1
13.	vanilloyl-*O*-hexose	$C_{14}H_{18}O_9$	329.0875	329.0879 (100), 269.0666 (7.8), 239.0557 (3.6), 209.0447 (34.4), 167.0337 (56.3), 152.0096 (5.0), 123.0435 (6.8), 108.0204 (4.1)	2.47	0.257	D1
14.	dihydrocaffeic acid-*O*-hexoside	$C_{15}H_{20}O_9$	343.1035	343.1035 (25.1), 181.0495 (100), 137.0593 (18.3), 109.0280 (5.3)	2.49	0.188	D2
15.	coumaric acid-*O*-hexoside 1	$C_{15}H_{18}O_8$	325.0930	163.0388 (100), 119.0487 (92.7)	2.51	0.336	D1
16.	caffeic acid–*O*-hexoside 1	$C_{15}H_{18}O_9$	341.0871	341.0875 (25.6), 281.0664 (1.9), 261.0430 (1.1), 221.0451 (1.9), 179.0338 (22.9), 161.0231 (100), 135.0437 (10.1), 133.0280 (27.1)	2.63	-0.836	D1
17.	*p*-hydroxyphenylacetic acid *O*-hexoside 1	$C_{14}H_{18}O_8$	313.0929	313.0933 (5.9), 269.1032 (2.4), 151.0387 (100), 107.0487 (0.4)	2.66	1.179	D1
18.	aesculetin-*O*-hexoside	$C_{15}H_{15}O_9$	339.0724	339.0721 (24.0), 177.0181 (100), 149.0230 (1.2), 133.0279 (10.1), 105.0329 (4.0), 89.0379 (2.2),	2.69	0.722	D1
19.	syringyl-*O*-hexose	$C_{15}H_{20}O_{10}$	359.0984	359.0980 (100), 299.0774 (4.4), 269.0667 (2.7), 239.0556 (22.0), 197.0446 (36.0), 182.0212 (4.4), 166.9975 (1.0), 153.0542 (1.9), 138.0305 (0.8), 121.0279 (2.7)	2.76	-1.142	D1
20.	4-hydroxybenzoic acid	$C_7H_6O_3$	137.0230	137.0229 (100), 109.0286 (5.7), 108.0201 (9.6), 93.0329 (16.9), 65.0380 (0.8)	2.84	-10.709	D1
21.	3-hydroxybenzoic acid	$C_7H_6O_3$	137.0230	137.0228 (23.8), 109.0278 (1.9), 93.0328 (100),	2.99	-11585	D1
22.	hydroxybenzoic acid-*O*-hexoside 2	$C_{13}H_{16}O_8$	299.0778	299.0767 (1.5), 137.0229 (100), 93.0329 (51.1)	2.99	-1.941	D1
23.	caffeic acid O-hexoside 2	$C_{15}H_{18}O_9$	341.0871	341.0877 (29.0), 179.0338 (100), 161.0233 (0.1), 135.0436 (73.7)	3.08	-2.068	D1
24.	*p*-coumaric acid	$C_9H_8O_3$	163.0389	163.0388 (11.5), 135.0437 (1.0), 119.0487 (100)	3.10	-7.835	B

25.	scopoletin *O*-hexoside	$C_{16}H_{18}O_9$	353.0878	353.0878 (91.6), 233.0450 (100), 205.0498 (58.0), 191.0341 (3.2), 163.0388 (6.3), 135.0436 (1.7), 85.0277 (1.8)	3.16	-0.015	D1
26.	dihydrocaffeic acid-O-hexoside	$C_{15}H_{20}O_9$	343.1035	343.1035 (25.1), 181.0495 (100), 137.0593 (18.3), 109.0280 (5.3)	3.19	0.451	D1
27.	*p*-hydroxyphenylacetic acid *O*-hexoside 2	$C_{14}H_{18}O_8$	313.0929	313.0927 (3.4), 151.0388 (9.9), 107.0486 (100)	3.30	0.030	D1
28.	coumaric acid-*O*-hexoside 2	$C_{15}H_{18}O_8$	325.0930	325.0930 (10.8), 163.0387 (15.2), 145.0280 (100), 119.0487 (7.7)	3.34	0.490	D1
29.	*m*-coumaric acid	$C_9H_8O_3$	163.0389	163.0384 (6.4), 135.0434 (0.4), 119.0487 (100)	3.35	-8.142	B
30.	vanillic acid-*O*-hexoside 2	$C_{14}H_{18}O_9$	329.0875	329.0869 (1.4), 209.0449 (1.2), 167.0337 (100), 123.0430 (22.2), 108.0198 (0.1)	3.39	-2.629	D1
31.	vanillyl alcohol-(acetyl)-hexoside	$C_{16}H_{22}O_9$	357.1191	357.1189 (5.4), 195.0654 (100), 153.0543 (7.1), 123.0436 (4.5)	3.41	-0.491	D1
32.	aesculetin	$C_9H_6O_4$	177.0193	177.0181 (100), 149.0231 (2.9), 133.0280 (20.1), 121.0277 (1.0), 105.0330 (11.0), 89.0380 (7.5)	3.45	-6.225	D1
33.	scopoletin O-hexoside isomer	$C_{16}H_{18}O_9$	353.0878	353.0877 (84.2), 283.0560 (23.6), 233.0450 (100), 205.0498 (41.1), 191.0342 (2.2), 163.0387 (2.1), 149.0224 (0.6), 135.0437 (1.1), 119.0486 (4.5)	3.48	-0.185	D1
34.	ferulic acid	$C_{10}H_{10}O_4$	193.0494	193.0497 (11.7), 178.0260 (16.9), 149.0594 (7.5), 134.0358 (100), 117.0331 (6.8), 89.0380 (3.4)	3.55	-4.932	B
35.	caffeic acid	$C_9H_8O_4$	179.0339	179.0339 (19.6), 135.0437 (100), 107.0489 (1.2), 93.0332 (0.2)	3.56	-6.267	B
36.	gentisic acid	$C_7H_6O_4$	153.0180	153.0180 (43.7), 109.0279 (100), 91.0172 (1.1)	3.67	-8.966	B
37.	vanillic acid-*O*-hexoside 3	$C_{14}H_{18}O_9$	329.0875	329.0881 (10.9), 209.0448 (10.7), 167.0336 (100), 123.0435 (34.2)	4.22	0.804	D1
38.	vanillin	$C_8H_8O_3$	151.0401	151.0388 (4.6), 136.0154 (3.6), 123.0436 (0.5), 107.0486 (100)	4.34	-8.193	B

39.	phenylethyl-*O*-pentosylhexoside (primeveroside)	$C_{20}H_{30}O_{12}$	461.1665	461.1666 (67.8), 415.1612 (100), 311.0988 (13.0), 293.0869 (1.3), 251.0771 (6.0), 221.0658 (6.6), 191.0552 (27.3), 179.0552 (0.7), 161.0446 (1.6), 149.0442 (57.5), 131.0335 (41.1), 89.0228 (64.8)	4.48	0.391	D1
40.	*o*-coumaric acid	$C_9H_8O_3$	163.0389	163.0388 (8.5), 135.0435 (0.4), 119.0487 (100)	4.55	-7.835	B
41.	coumaric acid *O*-hexoside 3	$C_{15}H_{18}O_8$	325.0929	325.0930 (10.6), 163.237 (81.3), 119.0487 (100),	4.70	1.992	D1
42.	phenylethyl-*O*-pentosylhexoside (primeveroside)	$C_{20}H_{30}O_{12}$	461.1665	461.1666 (8.8), 415.1612 (100), 311.0988 (2.1), 283.1182 (0.8), 191.0550 (5.7), 179.0554 (3.5), 161.0440 (2.3), 149.0442 (29.5), 131.0334 (11.7), 89.0228 (44.1)	4.75	0.457	D1
43.	vanillic acid	$C_8H_8O_4$	167.0338	167.0337 (37.9), 152.0101 (100), 139.0387 (3.1), 123.0436 (36.5), 108.0201 (52.8)	4.79	-7.436	B
44.	scopoletin – (caffeoyl)-hexoside	$C_{25}H_{26}O_{13}$	533.1301	533.1306 (100), 515.1012 (2.2), 473.1099 (9.2), 443.0983 (16.0), 413.0904 (3.4), 383.0772 (15.4), 353.0669 (18.2), 325.0721 (1.90, 297.765 (8.20, 191.0345 (0.9), 161.0235 (1.7), 135.0436 (0.6), 117.0332 (2.7), 85.0279 (0.5)	5.12	0.912	D1
45.	glansreginic acid *O*-hexoside	$C_{18}H_{28}O_{10}$	403.1609	403.1601 (66.6), 241.1078 (57.1), 223.0970 (100), 197.1175 (28.6), 179.1068 (40.3), 167.0700 (1.6), 149.0593 (6.8), 119.0334 (17.8), 101.0229 (21.2), 89.0228 (41.3), 71.0122 (39.7)	5.43	-2.158	D2
46.	digalloylcitramalic acid	$C_{19}H_{18}O_{14}$	469.0624	469.0515 (100), 317.0405 (6.8), 241.0349 (5.7), 169.0130 (97.9), 151.0024 (1.1), 125.0229 (63.2), 107.0123 (6.0), 95.0123 (3.8)	5.52	-23.171	D2
47.	salicylic acid	$C_7H_6O_3$	137.0230	137.0228 (11.9), 93.0330 (100), 65.0381 (1.0)	6.28	-11.585	B
48.	ferulic acid - (vanillyl)-hexoside	$C_{24}H_{26}O_{12}$	505.1352	505.1351 (93.1), 337.0931 (52.8), 193.0493 (12.6), 175.0390 (46.7), 167.0337 (100), 160.0153 (31.9), 152.0101 (63.0), 149.0590 (1.6), 108.0201 (36.0)	6.45	-0.157	D1
49.	gentisic acid-(feruloyl)-hexoside	$C_{23}H_{24}O_{12}$	491.1195	491.1197 (100), 315.0724 (14.3), 193.0500 (3.5), 153.0182 (12.9), 152.0101 (41.8)	6.71	0.449	D1

**Acyltartaric acids**							
50.	caftaric acid	$C_{13}H_{12}O_9$	311.0409	311.0415 (0.4), 179.0339 (15.4), 149.0078 (100), 135.0437 (12.3), 103.0022 (2.4), 87.0071 (13.4)	2.16	1.977	C
51.	caffeoyltartaric acid-hexoside-	$C_{19}H_{22}O_{14}$	473.0937	473.0930 (18.9), 341.0879 (82.6), 179.0339 (100), 161.0230 (1.1), 149.0078 (6.5), 135.0437 (91.5), 112.9869 (1.4), 87.0070(2.0),	2.28	-1.328	D2
52.	caftaric acid isomer	$C_{13}H_{12}O_9$	311.0409	311.0408 (0.4), 179.0339 (68.4), 149.0078 (100), 135.0437 (44.4), 112.9866 (0.5), 103.0022 (2.3), 87.0071 (13.5)	2.36	-0.177	D1
53.	cafeoyltartaric acid dimer	$C_{26}H_{23}O_{18}$	623.0892 [2M-H]$^-$	311.0410 (96.3), 179.0338 (100), 149.0078 (93.7), 135.0437 (82.3), 112.9868 (0.7), 103.0021 (2.9), 87.0071 (12.4), 59.0121 (2.2)	2.36	0.390	D2
54.	dicaffeoyltartaric acid 1	$C_{22}H_{20}O_{13}$	491.0831	491.0832 (69.6), 359.0771 (13.7), 315.0874 (3.8), 271.0973 (4.9), 255.0650 (3.4), 221.5004 (0.5), 191.0187 (0.6), 179.0338 (100), 161.0600 (4.2), 149.0594 (21.2), 135.0437 (89.6), 112.9865 (5.6), 87.0071 (11.6)	2.58	0.094	D2
55.	dicaffeoyltartaric acid 2	$C_{22}H_{20}O_{13}$	491.0831	491.0801 (31.4), 359.0774 (42.2), 315.0877 (4.0), 271.0985 (4.9), 255.0664 (3.8), 179.0338 (73.7), 149.0594 (29.9), 135.0437 (100), 112.9866 (2.7), 87.0071 (5.6)	2.79	-6.056	D2
56.	coumaroyltartaric acid	$C_{13}H_{12}O_8$	295.0459	295.0459 (1.4), 163.0388 (100), 149.0078 (11.2), 119.0487 (31.3), 112.9865 (7.4), 103.0022 (3.3), 87.0071 (8.0)	3.10	-0.239	D2
57.	feruloyltartaric acid	$C_{14}H_{14}O_9$	325.0565	325.0566 (2.1), 193.0497 (100), 178.0263 (2.0), 165.0538 (0.3), 149.0078 (13.9), 134.035 (50.5), 12.9864 (10.5), 87.0071 (9.0)	3.55	-0.139	D2
**Citric/isocitric acid esters (Acylcitric acids)**							
58.	caffeoylcitric/isocitric acid 1	$C_{15}H_{14}O_{10}$	353.0514	353.0509 (2.2), 309.0618 (4.5), 191.0188 (98.7), 179.0338 (2.7), 161.0230 (9.2), 147.0285 (31.2), 135.0437 (2.3), 133.0281 (3.5), 103.0021 (0.9), 85.0279 (100)	2.41	-1.472	D1

59.	caffeoylcitric/isocitric acid 2	$C_{15}H_{14}O_{10}$	353.0514	353.0524 (1.2), 309.0606 (3.2), 191.0187 (100), 179.0340 (2.2), 161.0234 (11.4), 147.0285 (16.1), 135.0436 (3.1), 133.0282 (5.5), 103.0019 (0.9), 85.0278 (76.6)	2.76	2.776	D1
60.	caffeoylcitric/isocitric acid 3	$C_{15}H_{14}O_{10}$	353.0514	353.0518 (2.3), 309.0625 (3.3), 191.0187 (100), 179.0330 (0.7), 161.0233 (3.3), 147.0285 (12.4), 135.0436 (1.2), 133.0279 (1.9), 103.0021 (0.5), 85.0278 (76.1)	2.90	2.267	D1
61.	caffeoylcitric/isocitric acid 4	$C_{15}H_{14}O_{10}$	353.0514	353.0518 (2.8), 309.0613 (0.7), 191.0187 (100), 179.0335 (4.2), 161.0233 (3.3), 147.0285 (5.2), 135.0437 (7.3), 103.0021 (0.8), 85.0278 (69.5)	3.28	1.134	D1
62.	caffeoylcitric/isocitric acid 5	$C_{15}H_{14}O_{10}$	353.0514	353.0522 (4.2), 309.0628 (0.5), 191.0187 (100), 179.0337 (6.9), 161.0232 (19.2), 147.0285 (17.9), 135.0436 (13.2), 133.0281 (6.0), 85.0278 (77.3)	3.95	2.267	D1
63.	coumaroylcitric/isocitri c acid 1	$C_{15}H_{14}O_9$	337.0565	337.0572 (3.4) 191.0188 (32.7), 163.0388 (100), 147.0285 (1.3), 135.0434 (0.7), 129.0178 (23.5), 119.0486 (38.1), 85.0278 (51.1)	4.16	1.943	D1
64.	caffeoylcitric/isocitric acid 6	$C_{15}H_{14}O_{10}$	353.0514	353.0517 (4.2), 309.0636 (0.7), 191.0188 (68.9), 179.0340 (19.5), 161.0232 (29.4), 147.0285 (46.8), 135.0437 (21.2), 133.0281 (9.7), 85.0278 (100)	4.29	8.879	D1
65.	coumaroylcitric/isocitri c acid 2	$C_{15}H_{14}O_9$	337.0565	337.0564 (23.0) 191.0188 (55.8), 163.0389 (100), 147.0283 (6.7), 145.0281 (70.7), 131.0658 (38.3), 129.0178 (39.5), 119.0487 (100)	4.85	-0.401	D2
66.	coumaroylcitric/isocitri c acid 3	$C_{15}H_{14}O_9$	337.0565	337.0562 (14.9) 293.0653 (0.1), 191.0188 (75.8), 163.0387 (15.5), 147.0285 (19.9), 145.0281 (33.2), 129.0179 (8.5), 119.0487 (14.4), 103.0018 (0.7), 85.0279 (100)	5.26	-0.876	D2
67.	feruloylcitric/isocitric acid 1	$C_{16}H_{16}O_{10}$	367.0671	367.0655 (34.8), 323.0770 (2.5), 261.0767 (49.6), 193.0497 (50.9), 191.0189 (28.1), 178.0263 (3.4), 175.0390 (39.6),173.0079 (0.8), 149.0595 (16.4), 147.0285 (8.8), 134.0359 (46.9), 129.0179 (46.5), 103.0019 (0.7), 85.0279 (100)	5.30	0.028	D2

68.	feruloylcitric/isocitric acid 2	$C_{16}H_{16}O_{10}$	367.0671	367.0667 (18.7), 261.0768 (20.4), 193.0497 (14.5), 191.0187 (41.1), 175.0390 (26.0), 173.0081 (5.2), 149.0596 (4.4), 147.0285 (42.5), 134.0359 (23.2), 129.0179 (13.0), 85.0278 (100)	5.63	-0.899	D2
**Gallotannins**							
69.	galloyl-*O*-hexoside 1	$C_{13}H_{16}O_{10}$	331.0671	331.0672 (100), 113.0567 (6.1), 169.0126 (3.8), 168.0052 (32.3), 125.0229 (31.6), 107.0121 (1.0)	1.22	0.484	D1
70.	galloyl-*O*-hexoside 2	$C_{13}H_{16}O_{10}$	331.0671	331.0676 (10.4), 169.0130 (100), 125.0229 (40.4)	1.58	1.692	D1
71.	gallocatechin	$C_{15}H_{14}O_7$	305.0667	305.0668 (90.8), 287.0550 (0.7), 261.0770 (9.3), 219.0657 (17.4), 179.0340 (27.6), 167.0338 (20.6), 137.0230 (28.6), 125.0229 (100), 109.0279 (17.1)	1.80	0.407	D1
72.	digalloyl-O-hexose	$C_{20}H_{20}O_{14}$	483.0780	483.0781 (100), 331.0675 (5.2), 313.0569 (16.0), 271.0460 (43.4), 211.0241 (12.0), 169.0131 (37.1), 151.0024 (2.1), 125.0229 (29.0), 107.0123 (4.5)	2.99	0.065	D1
73.	digallic acid	$C_{14}H_{10}O_9$	321.0252	321.0255 (2.7), 169.0130 (100), 125.0227 (42.5), 97.0278 (1.8)	3.10	1.043	D1
74.	methylgallate	$C_8H_8O_5$	183.0299	183.0288 (100), 168.0052 (11.7), 140.0102 (10.9) 111.0072 (6.4), 83.0122 (0.8)	3.16	-6.101	D1
75.	tetragalloyl-hexoside	$C_{34}H_{28}O_{22}$	787.1000	787.1011 (100), 635.0829 (1.9), 617.0780 (7.3), 465.0674 (34.5), 403.0668 1.90, 313.0572 (15.8), 25.0456 (7.9), 169.0131 (77.8), 15.0023 (2.7), 1390022 (5.7), 125.0229 (70.3), 107.0124 (6.9), 95.012 (5.6)	4.94	1.518	D1
76.	ellagic acid	$C_{14}H_6O_8$	300.9990	300.9987 100), 257.0085 (1.0), 229.0137 (3.4), 217.0137 (0.9), 201.0183 (2.4), 185.0231 (2.9), 173.0229 (2.6), 145.0281 (2.7), 129.0328 (0.8)	5.01	-1.098	B
**Flavonoids**							
77.	eriodyctiol *O*-hexoside	$C_{21}H_{22}O_{11}$	449.1089	449.1092 (100), 287.0561 (26.8), 269.0455 (37.4), 259.0611 (46.4), 243.0661 (2.7), 225.0555 (2.2), 201.0545 (2.0),	4.03	0.635	D1

				178.9975 (10.5), 151.0022 (13.2), 125.0229 (47.7), 207.0122 (4.2)			
78.	myricitin 3-*O*-pentosylhexoside	$C_{26}H_{28}O_{17}$	611.1254	611.1258 (90.0), 317.0277 (12.1), 316.0223 (100), 287.0197 (15.6), 271.0247 (25.3), 259.0248 (6.5), 242.026 (3.6), 227.0344 (0.3), 214.0265 (5.4), 178.9975 (3.5)1561.0024 (3.0)	4.18	0.683	D1
79.	myricetin 3-*O*-rutinoside	$C_{27}H_{30}O_{17}$	625.1410	625.1417 (100), 317.0288 (22.5), 316.0224 (95.7), 287.0199 (14.9), 271.0248 (24.2), 259.0255 (5.3), 242.0214 (4.30, 214.0264 (5.7), 178.9972 (4.3), 151.0023 (4.5), 107.0122 (2.3)	4.48	1.116	D1
80.	quercetin 3-*O*-pentosylhexoside	$C_{26}H_{28}O_{16}$	595.1305	595.1310 (79.5), 445.089 (0.5), 325.0338 (0.1), 301.0330 (12.8), 300.0273 (100), 271.0247 (41.5), 255.0296 (17.3), 243.0294 (9.2), 227.0342 (2.6), 178.9975 (2.3), 163.0023 (0.8), 151.0024 (2.7), 121.0275 (0.3), 107.0123 (1.4)	4.71	-0.324	D1
81.	myricetin *O*-pentoside	$C_{20}H_{18}O_{12}$	449.0726	449.0727 (100), 317.0277 (14.1), 316.0224 (98.4), 2870190 (16.1), 271.0248 (24.2), 259.0248 (5.6), 242.0219 (4.2), 214.0266 (6.4), 178.9974 (2.5), 151.0023 (3.1), 107.0122 (1.2)	4.96	0.381	D1
82.	rutin	$C_{27}H_{30}O_{16}$	609.1464	609.1467 (100), 301.0349 (38.6), 300.0275 (68.3), 271.0248 (38.6), 255.0297 (19.1), 243.0294 (9.1), 227.0344 (2.6), 211.0399 (1.0), 178.9974 (2.6), 175.0381 (02), 163.0026 (1.7), 151.0023 (6.6), 121.0279 (1.2), 107.0123 (2.1)	5.08	2.121	C
83.	myricetin *O*-rhamnoside (myricitrin)	$C_{21}H_{20}O_{12}$	463.0885	463.0887 (100), 317.0290 (26.8), 316.0224 (96.2), 287.0199 (15.7), 271.0247 (26.3), 259.0246 (6.4), 242.0214 (4.4), 214.0265 (5.7), 178.9973 (3.7), 151.0022 (6.3), 137.0228 (2.2), 107.0122 (2.1)	5.11	0.974	C
84.	hyperoside	$C_{21}H_{20}O_{12}$	463.0885	463.0886 (100), 301.0348 (35.3), 300.0276 (73.2), 271.0249 (36.2), 255.0296 (16.2), 211.0389 (0.5), 199.0385 (0.5), 178.9981 (2.5), 163.0027 (1.4), 151.0024 (5.8), 121.0277 (1.3), 107.0123 (1.8)	5.19	0.779	C
85.	quercetin *O*-hexuronide	$C_{22}H_{22}O_{12}$	477.1039	477.0677 (73.5), 301.0353 (100), 283.0247 (1.3), 273.0409 (1.2), 255.030 (3.5), 227.0341 (2.2), 211.0398 (1.4), 178.9976 (8.5), 163.0027 (3.8), 151.0024 (22.3), 121.0280 (6.7), 107.0123 (8.5)	5.23	0.600	D1

86.	kaempferol *O*-pentosylhexoside	$C_{26}H_{28}O_{15}$	579.1355	579.1359 (100), 429.0831 (1.5), 285.0391 (22.1), 284.0328 (94.3), 255.0298 (48.2), 227.0347 (32.7), 211.0391 (1.6), 178.9980 (0.9), 151.0025 (2.1), 107.0121 (2.0)	5.27	2.484	D1
87.	isoquercitrin	$C_{21}H_{20}O_{12}$	463.0885	463.0886 (100), 301.0351 (38.5), 300.0276 (70.0), 255.0298 (14.9), 243.0297 (9.3), 227.0339 (2.6), 201.0401 (0.5), 178.9977 (2.4), 163.0022 (1.7), 151.0021 (6.2), 121.0279 (0.7), 107.0121 (2.1)	5.30	0.779	C
88.	myricetin methylether *O*-hexoside	$C_{22}H_{22}O_{13}$	493.0988	493.0964 (100), 331.0450 (31.8) 330.0380 (68.0), 315.0150 (16.5), 287.0194 (16.0), 271.0251 (7.4), 259.0243 (9.1), 243.0302 (4.6), 231.0295 (0.7), 215.0348 (4.2), 203.0345 (4.3), 185.1174 (12.9), 178.9977 (1.6), 163.0027 (1.0), 151.0027 (4.3), 107.0122 (1.1)	5.35	1.229	D1
89.	phloretin 3′, 5–diC-hexoside	$C_{27}H_{34}O_{15}$	597.18249	597.1840 (100), 507.1487 (2.6), 477.1412 (57.0), 417.1197 (10.0), 387.1085 (47.0), 357.0984 (58.9), 315.0878 (50.0), 273.0769 (3.1), 179.0342 (3.7), 167.0335 (18.9), 125.0228 (8.2), 123.0436 (13.4), 119.0485 (6.4), 81.0328 (10.3)	5.37	2.456	D1
90.	kaempferol *O*-deoxyhexosyl-*O*-hexoside	$C_{27}H_{30}O_{15}$	593.1512	593.1518 (100), 285.0400 (27.2), 284.0327 (58.4), 255.0299 (31.6), 227.0348 (22.2). 211.0386 (0.7), 151.0024 (2.4), 107.0124 (0.7)	5.41	1.006	D1
91.	quercetin 3-*O*-pentoside	$C_{20}H_{18}O_{11}$	433.0776	433.0777 (100), 301.0342 (26.1), 300.075 (98.2), 271.0248 (37.5), 255.0296 (16.7), 227.0344 (2.7), 211.0405 (0.6), 178.9970 (1.0), 163.0025 (0.7), 151.0020 (3.6), 107.0117 (1.8)	5.64	0.220	D1
92.	kaempferol 3-*O*-rutinoside	$C_{27}H_{30}O_{15}$	593.1512	593.1516 (100), 285.0400 (27.2), 284.0327 (58.4), 255.0299 (31.6), 227.0348 (22.2), 211.0386 (0.7), 151.0024 (2.4), 107.0124 (0.7)	5.64	0.703	C
93.	phloretin C-hexoside	$C_{21}H_{24}O_{10}$	435.1297	435.1304 (18.1), 345.0978 (23.3), 315.0876 (100), 273.0766 (7.4), 179.0339 (4.1), 167.0337 (23.3), 125.0229 (11.0), 123.0436 (8.8), 119.0487 (5.3), 81.0329 (6.3)	5.74	1.677	D1
94.	isorhamnetin 3-*O*-rutinoside	$C_{28}H_{32}O_{17}$	623.1618	623.1622 (100), 315.0511 (85.3), 300.0270 (14.2), 299.0194 (14.6), 271.0247 (25.4), 255.0301 (10.3), 243.0296 (0.7), 227.0312 (3.8), 199.0397 (4.0), 151.0020 (2.6)	5.78	2.025	C

95.	kaempferol glucoside	3-*O*-	$C_{21}H_{19}O_{11}$	447.0934	447.0934 (100), 327.0515 (1.7), 285.0402 (23.7), 284.0327 (54.1), 255.0299 (39.0), 227.0346 (35.9), 211.0401 (0.5), 151.0022 (0.8), 107.0121 (0.5)	5.88	0.348	C
96.	isorhamnetin glucoside	3-*O*-	$C_{22}H_{22}O_{12}$	477.1044	477.1039 (100), 357.0645 (0.7), 315.0448 (1.0), 314.0434 (47.8), 299.0196 (2.7), 285.0408 (6.1), 271.0249 (19.4), 257.0451 (3.6), 243.0297 (18.3), 227.0347 (2.5), 215.0357 (2.9), 199.0394 (3.3), 178.9979 (0.5), 151.0022 (2.9)	6.03	0.002	C
97.	naringenin hexoside	7-*O*-	$C_{21}H_{20}O_{10}$	431.0984	433.1158 (4.6), 271.0611 (100), 151.0023 (18.9), 119.0487 (21.4), 107.0123 (7.1)	6.06	4.202	D1
98.	kaempferol pentoside	3-O-	$C_{20}H_{18}O_{10}$	417.0827	417.0829 (100), 285.0395 (17.7), 284.0327 (62.3), 256.0299 (40.2), 227.0346 (38.9), 21.0397 (0.6), 151.0025 (0.7), 107.0124 (0.4)	6.07	0.336	D1
99.	eriodyctiol		$C_{15}H_{12}O_6$	287.0561	287.0558 (67.5), 259.0611 (100), 243.0322 (19.0), 227.1279 (4.2), 215.0704 (5.5), 178.9968 (9.6), 151.0024 (12.2), 125.0229 (96.2), 107.0122 (7.0)	6.29	-1.119	B
100.	myricetin		$C_{15}H_{10}O_8$	317.0303	317.0302 (100), 299.0187 (0.9), 289.0338 (0.3), 271.0250 (1.0), 243.0298 (30.3), 227.0336 (0.3), 178.9977 (24.5), 165.0181 (1.5), 151.0025 (29.8), 137.0231 (22.9), 107.0123 (10.8)	6.29	-0.159	B
101.	luteolin caffeoylhexoside	7-*O*-	$C_{30}H_{26}O_{14}$	609.1250	609.1252 (100), 447.0949 (4.2), 285.0403 (61.3), 255.0294 (19.8), 227.0315 (12.0), 179.0340 (12.7), 161.0231 (26.1), 151.0022 (2.6), 135.04378 (14.8), 133.0281 (10.5), 107.0122 (0.8)	7.01	0.331	D1
102.	quercetin		$C_{15}H_{10}O_7$	301.0354	301.0353 (100), 273.0409 (2.8), 257.0470 (1.7), 243.0294 (0.3), 229.0503 (1.3), 211.0399 (0.5), 178.9975 (21.0), 161.0230 (0.3), 151.0023 (45.6), 121.0280 (13.1), 107.0123 (15.8),	7.61	-0.219	B
103.	isorhamnetin		$C_{16}H_{12}O_7$	315.0512	315.0510 (63.7), 300.0274 (100), 271.0246 (35.0), 255.0296 (13.5), 243.0097 (9.3), 227.0348 (1.8), 199.0393 (1.1), 151.0027 (0.5), 135.0075 (1.0), 107.0127 (0.5)	8.09	-0.114	B

104.	myricetin dimethyl ether	$C_{17}H_{14}O_8$	345.0616	345.0616 (89.9), 330.0383 (44.4), 315.0147 (10.0), 287.0197 (46.6), 259.0243 (7.5), 243.0296 (3.7), 231.0287 (3.4), 215.0340 (3.6), 203.0331 (1.6), 187.0396 (1.5), 151.0019 (1.6)	8.11	-0.118	D1
105.	naringenin	$C_{15}H_{12}O_5$	271.0612	271.0612 (100), 227.0703 (0.5), 151.0023 (67.6), 119.0487 (53.2), 107.0122 (15.2)	8.58	-0.025	B
106.	kaempferol dimethyl ether *O*-hexoside	$C_{31}H_{22}O_8$	521.1242	521.1215 (4.0), 475.1250 (10.6), 355.0798 (0.5), 313.0718 (100), 298.0480 (11.7), 283.0247 (27.5), 255.0295 (18.6), 241.0511 (0.5), 226.0290 (0.5)	8.79	-5.125	D1
107.	kaempferol	$C_{15}H_{10}O_6$	285.0405	285.0403 (100), 257.0454 (0.7), 229.0499 (1.0), 211.0391 (1.1), 163.0026 (0.3), 151.0023 (1.3), 107.0121 (1.3)	8.84	-0.531	B
108.	kaempferol methyl ether (kaempferide)	$C_{16}H_{12}O_6$	299.0561	299.0560 (64.5), 284.0324 (100), 255.0296 (46.9), 227.0344 (34.0), 211.0390 (1.8), 183.0440 (1.7), 151.0018 (0.5), 135.0069 (1.4), 132.0199 (1.2), 107.0118 (1.2)	9.33	-0.473	D1
109.	quercetin dimethyl ether	$C_{17}H_{14}O_7$	329.0677	329.0668 (100), 314.0435 (44.3), 299.0198 (69.2), 271.0248 (51.4), 257.0453 (0.8), 227.0346 (4.7), 199.0389 (5.6), 151.0023 (1.6), 133.0283 (0.5), 107.0121 (1.6)	9.61	0.377	D1
110.	myricetin trimethyl ether	$C_{18}H_{16}O_8$	359.0772	359.0775 (68.7), 344.0536 (100), 329.0302 (84.8), 314.0078 (1.0), 301.0352 (9.9), 286.0122 (8.0), 273.0403 (12.2), 258.0168 (10.3), 230.0218 (12.4), 202.0267 (5.2), 165.0180	10.68	0.416	D1
111.	kaempferol dimethyl ether	$C_{17}H_{14}O_6$	313.0718	313.0719 (77.0), 298.0483 (100), 283.0247 (49.7), 255.0297 40.8), 227.0340 (2.0), 211.0388 (3.2), 183.0440 (3.5), 151.0024 (1.33), 117.0330 (0.6),107.0121 (0.5)	12.30	0.347	D1
**Other compounds**							
112.	bergenin *O*-coumaric acid	$C_{23}H_{22}O_{11}$	473.1089	473.1106 (8.1), 327.0723 (19.4), 309.0623 (6.2), 265.0714 (43.4), 187.0236 (44.1), 163.0387 21.9), 129.0180 (8.7), 121.0279 (68.5), 111.0071 (100)	8.03	3.583	D1

Assays for Total Phenolic and Flavonoid Contents

The total phenolic content was determined by employing the methods given in the literature with some modification. Sample solution (0.25 mL) was mixed with diluted Folin–Ciocalteu reagent (1 mL, 1:9, v/v) and shaken vigorously. After 3 min, Na_2CO_3 solution (0.75 mL, 1%) was added and the sample absorbance was read at 760 nm after a 2 h incubation at room temperature. Gallic acid was used as a standard (0.01-01 mg/ml). The total phenolic content was expressed as milligrams of gallic acid equivalents (mg GAE/g extract) [77]

The total flavonoid content was determined using the $AlCl_3$ method. Briefly, sample solution (1 mL) was mixed with the same volume of aluminum trichloride (2%) in methanol. Similarly, a blank was prepared by adding sample solution (1 mL) to methanol (1 mL) without $AlCl_3$. The sample and blank absorbances were read at 415 nm after a 10 min incubation at room temperature. The absorbance of the blank was subtracted from that of the sample. Rutin was used as a reference standard (0.02-0.01 mg/ml) and the total flavonoid content was expressed as milligrams of rutin equivalents (mg RE/g extract) [85]

Determination Enzyme Inhibitory Effects

For Cholinesterase (ChE) inhibitory activity assay: Sample solution (was mixed with DTNB (5,5-dithio-bis(2-nitrobenzoic) acid, Sigma, St. Louis, MO, USA) (125 μL) and AChE (acetylcholinesterase (Electric ell acetylcholinesterase, Type-VI-S, EC 3.1.1.7,Sigma)), or BChE (butyrylcholinesterase (horse serum butyrylcholinesterase, EC 3.1.1.8, Sigma)) solution (25 μL) in Tris–HCl buffer (pH 8.0) in a 96-well microplate and incubated for 15 min at 25 °C. The reaction was then initiated with the addition of acetylthiocholine iodide (ATCI, Sigma) or butyrylthiocholine chloride (BTCl, Sigma) (25 μL). Similarly, a blank was prepared by adding sample solution to all reaction reagents without enzyme (AChE or BChE) solution. The sample and blank absorbances were read at 405 nm after 10 min incubation at 25 °C. The absorbance of the blank was subtracted from that of the sample. Galanthamine was used as a standard (1-5 μg/ml) and the cholinesterase inhibitory activity was expressed as galanthamine equivalents (mg GALAE/g extract) [86].

For Tyrosinase inhibitory activity assay: Sample solution was mixed with tyrosinase solution (40 μL, Sigma) and phosphate buffer (100 μL, pH 6.8) in a 96-well microplate and incubated for 15 min at 25 °C. The reaction was then initiated with the addition of L-DOPA (40 μL, Sigma). Similarly, a blank was prepared by adding sample solution to all reaction reagents without enzyme (tyrosinase) solution. The sample and blank absorbances were read at 492 nm after a 10 min incubation at 25 °C. The absorbance of the blank was subtracted from that of the sample. Kojic acid was used as a standard (0.1-1 mg/ml) and the tyrosinase inhibitory activity was expressed as kojic acid equivalents (mg KAE/g extract) [87].

For α-amylase inhibitory activity assay: Sample solution was mixed with α-amylase solution (ex-porcine pancreas, EC 3.2.1.1, Sigma) (50 μL) in phosphate buffer

(pH 6.9 with 6 mM sodium chloride) in a 96-well microplate and incubated for 10 min at 37 °C. After pre-incubation, the reaction was initiated with the addition of starch solution (50 μL, 0.05%). Similarly, a blank was prepared by adding sample solution to all reaction reagents without enzyme (α-amylase) solution. The reaction mixture was incubated 10 min at 37 °C. The reaction was then stopped with the addition of HCl (25 μL, 1 M). This was followed by addition of the iodine-potassium iodide solution (100 μL). The sample and blank absorbances were read at 630 nm. The absorbance of the blank was subtracted from that of the sample. Acarbose was used as a standard (0.1-1 mg/ml) and the α-amylase inhibitory activity was expressed as acarbose equivalents (mmol ACE/g extract) [88].

For α-glucosidase inhibitory activity assay: Sample solution was mixed with glutathione (50 μL), α-glucosidase solution (from Saccharomyces cerevisiae, EC 3.2.1.20, Sigma) (50 μL) in phosphate buffer (pH 6.8) and PNPG (4-N-trophenyl-α-D-glucopyranoside, Sigma) (50 μL) in a 96-well microplate and incubated for 15 min at 37 °C. Similarly, a blank was prepared by adding sample solution to all reaction reagents without enzyme (α-glucosidase) solution. The reaction was then stopped with the addition of sodium carbonate (50 μL, 0.2 M). The sample and blank absorbances were read at 400 nm. The absorbance of the blank was subtracted from that of the sample. Acarbose was used as a standard (0.1-1 mg/ml) and the α-glucosidase inhibitory activity was expressed as acarbose equivalents (mmol ACE/g extract) [89].

References

77. Slinkard, K.; Singleton, V.L. Total phenol analysis: Automation and comparison with manual methods. *Am. J. Enol. Vitic.* **1977**, *28*, 49–55.
85. Arvouet-Grand, A.; Vennat, B.; Pourrat, A.; Legret, P. Standardization of propolis extract and identification of principal constituents. *J. Pharm. Bel.g* **1994**, 49, 462–468.
86. Ellman, G.L.; Courtney, K.D.; Andres, V., Jr.; Featherstone, R.M. A new and rapid colorimetric determination of acetylcholinesterase activity. *Biochem. Pharmacol.* **1961**, *7*, 88–95.
87. Masuda, T.; Yamashita, D.; Takeda, Y.; Yonemori, S. Screening for tyrosinase inhibitors among extracts of seashore plants and identification of potent inhibitors from Garcinia subelliptica. *Biosci. Biotechnol. Biochem.* **2005**, *69*, 197–201.
88. Šafašík, I. Rapid Detection of Alpha-Amylase Inhibitors. *J. Enzym. Inhib.* **1990**, *3*, 245–247.
89. Ting, L.; Zhang, X.-D.; Song, Y.-W.; Liu, J.-W. A microplate-based screening method for alpha-glucosidase inhibitors. *Chin. J. Clin. Pharmacol. Ther.* **2005**, *10*, 1128.
